# Experimental study on I/II/III mixed mode fracture characteristics of a combined rock mass under creep loading

**DOI:** 10.1038/s41598-024-61056-9

**Published:** 2024-05-06

**Authors:** Shuai Li, Chao Zheng, Peng Li, Shuo Zhang

**Affiliations:** School of Civil and Transportation Engineering, Henan University of Urban Construction, Pingdingshan, People’s Republic of China

**Keywords:** Sandstone, Two sets of persistent joints, Creep experiment, Acoustic emission (AE), Fracture pattern, Engineering, Civil engineering

## Abstract

I/II/III mixed mode fractures of intersecting joint fissures often occur in natural rock masses, and jointed rock masses are prone to rockbursts in deep underground engineering when subjected to long-term crustal stresses. However, most studies of the mechanical mechanisms of these intersected joints have been conducted by simplifying two-dimensional joint model tests. Furthermore, the fracture mechanisms of two-dimensional intersected joints under tension and compression are completely different from those of three-dimensional joints. This paper presents a novel prefabricated specimen with combinations of intersecting joints capable of detecting the failure behaviours of rock I/II/III mixed mode fractures under creep loading. Uniaxial compression and multistage creep tests are performed on prefabricated sandstone specimens with intersecting joints of 0°/0°, 0°/30°, 0°/60°, and 0°/90°. The experimental results show that with the increase in the number of prefabricated intersecting joints, the uniaxial compressive strength and elastic modulus values of the sandstone specimens gradually decrease. In addition, the sandstone specimens experience relatively few AE events and minor axial strain variations in the first creep stage and the second creep stage of the multistage creep test. The axial strain increases sharply due to the sharp increase in the number of AE events in the third creep stage. The 0°/60° sandstone specimen undergoes accelerated creep failure, resulting in mixed X-shaped tensile‒shear rupture. The RA value is high based on the quantification of the creeping cracks using the acoustic emission parameters of the rise angle (RA) and average frequency (AF). The AF values of the 0°/0°, 0°/30°, and 0°/90° sandstone specimens are high. The experimental results show that a larger joint intersection angle leads to greater mutual restraints and greater effects of prefabricated crack propagation in the rock specimens, thus increasing the final failure strength. Finally, based on the acoustic emission count, a characteristic variable D suitable for characterizing the creep damage evolution of a joint rock mass is established. The findings of this paper can facilitate an effective understanding of the creep effect of I/II/III mixed mode fracture and its micromechanism. The research results will have a certain reference value for the detection and risk mitigation of instantaneous and time-delayed rockbursts.

## Introduction

**Figure 1:**
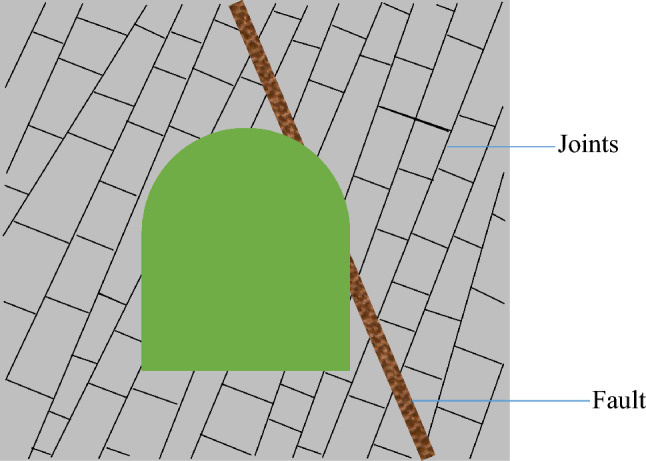
A two-dimensional projection of tunnel with the heavily jointed rock mass.

When a tunnel engineering project is implemented under soft rock mass conditions, unexpected disasters such as landslides, rockbursts, and large deformations may occur^1–3^. Soft rock masses such as sandstone often contain multiple groups of mutually intersecting joints, exhibiting complex structural characteristics^4^, as shown in Fig. 1. Under the long-term action of high crustal stresses in deep rock masses, the existing joint surface or fault undergoes shear slip, generating new cracks with time^5^. Importantly, due to the existence of open cracks and shear‒slip cracks, the strength of the original rock mass is reduced by 10–50%^6^. Thus, the mechanical behaviours of intersected joints must be further studied to effectively guide support design and stability analysis for rock mass engineering.

### Types of cracks

**Figure 2:**
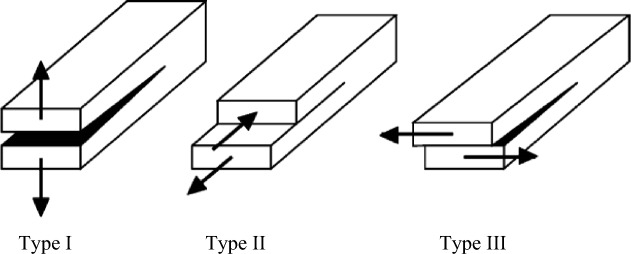
Three basic types of crack propagation (Chang et al., 2002).

Fracture mechanics adequately account for the mechanical behaviours of these jointed rock masses. In classic theory, cracks are divided into three basic types according to the geometric relationship between the external force action line and the crack surface^7^, as shown in Fig. 2: type I fractures (the tensile opening mode) are subjected to tensile stress perpendicular to their surfaces; type II fractures (the in-plane sliding/shear mode, or the in-plane shear type) are subjected to shear stress parallel to their surfaces and perpendicular to their leading edges; and type III fractures (the tearing/out-of-plane mode, also known as the out-of-plane shear type) are subjected to shear stress parallel to their surfaces and their leading edges. Ayatollahi et al.^8^ studied the type I fracture toughness values of white marble centre-crack disc samples with different radii. The test results show that the apparent fracture toughness values of the samples increase with increasing size. By conducting a static loading test on semicircular Brazilian disk (SCB) and Brazilian disk specimens with vertical prefabricated cracks, Omidimanesh et al.^9^ concluded that the type I fracture toughness (K_IC_) is not affected by the prefabricated vertical crack length and compared the test results with the numerical model results established by PFC2D. Bahrami et al.^10^ conducted a type II fracture toughness test using double-edged grooved Brazilian disk (DNBD) specimens and compared the predicted results of the theoretical model with the experimental data based on the mathematical model established with consideration of the fracture process zone and energy release rate (ERR). Bahrami et al.^11^ discussed the true type II fracture toughness values of rocks via the double-sided notched Brazilian disk test (DNBD). Theoretical analysis revealed that large compressive stress values in DNBD samples significantly promote the formation of true type II fractures. Simultaneously, three types of rock (limestone, marble, and granite) were tested with the new method for two true type II fractures with different toughness and length values. Aliha et al.^12^ investigated the fracture toughness values of white marble specimens with pure type I and pure type II fractures. The average type II fracture toughness (K_IIc_) is significantly greater than the type I fracture toughness (K_Ic_). The type II fracture toughness test data are estimated using generalized maximum tangential stress theory and type I fracture toughness test data. Khan et al.^13^ investigated the effects of diameter, thickness, fracture length and fracture type on the fracture toughness measurements using semicircular limestone samples under three-point bending for the Brazilian disc test. The results show that the specimen diameter and crack type strongly influence the fracture toughness. However, the effects of the loading rate, crack size and specimen thickness on the fracture toughness seem to be negligible. Pietras et al.^14^ measured the type III fracture toughness with a three-point curved beam, and the test results showed that the type III fracture is obvious and sensitive to notch depth. The type III fracture toughness is greater than the type I fracture toughness. Although the above studies are focused on type I tensile failure, type II shear failure, or type I–II tensile shear mixed failure, there are few studies on type III tear failure and type I–II–III mixed failure, mainly due to the complexity of laboratory testing, which cannot guarantee the generation of a specific failure mode.

### Time dependency of rocks

The damage and rupture of rock are time dependent, and some scholars have used continuum materials, such as intact rock and rock masses, for research. Paraskevopoulou et al.^15^ conducted relaxation tests on two types of limestone, measuring stress‒time responses at different load levels and revealing three distinct stages of stress relaxation. The rate of stress relaxation in the first stage gradually decreases, the rate of stress relaxation in the second stage tends to be constant, and stress relaxation in the third stage no longer occurs. Guessous et al.^16^ carried out a radial prestrain axial compression creep test of rock salt, and the test results showed that the creep response of salt is strongly affected by preloading in the short term. However, this effect decreases with increasing plastic deformation, suggesting that large creep strains may eventually lead to a complete loss of preloaded memory. Schapery^17^ developed constitutive equations through nonequilibrium thermodynamics, rate process theory, and viscoelastic fracture mechanics, which represent viscoelasticity, viscoplasticity, growth damage, and ageing, respectively. The change in damage is considered an internal state variable, and the expressions of the scalar state variable and tensor state variable are compared. Bérest et al.^18^ conducted a creep test on rock salt under a 0.1 MPa uniaxial load in an underground mine chamber and crushed the salt sample under a 0.24 MPa uniaxial load. After 6 months, the typical steady-state strain rate reaches − 2.4 × 10^–12^ s^−1^. Hamza^19^ conducted multistage uniaxial and triaxial creep tests on the ageing characteristics of argillous siltstone. The results show that the instantaneous strain and creep strain are proportional to the deviatoric stress and confining pressure. The relationship between axial strain and time is successfully fitted to the Burgers creep model. Roberts et al.^20^ applied triaxial cyclic loading creep tests on rock salt to simulate periodic compressed air energy storage in salt caverns, and cyclic triaxial creep tests were conducted under different load paths, including compression–stretching. Sinkala et al.^21^ suggested that intact rock specimens are susceptible to rockbursts and that the rockbursts are not transient rock mass failures caused by increased mining stress; the scholars proposed a creep damage model capable of accounting for time-sensitive rockbursts. However, natural rock masses have anisotropic and discontinuous joints, and the deformation of rock masses is greatly affected by the joints in tunnels excavated in these rock masses. Joint opening, displacement and/or development deformation often occur over long periods. Hence, it is difficult to evaluate the time-dependent deformation of a jointed rock mass.

This paper presents a method suitable for rockburst testing with mixed type I–II–III failure modes and evaluates the differences in crack modes under different intersecting joint angles (φ) by conducting uniaxial compression tests and multistep creep tests of sandstone with intersecting joints (joint angles φ = 0°, 30°, 60°, and 90°). Then, acoustic emission (AE) parameters are used to study the spatiotemporal deformation damage evolution characteristics of sandstone samples with intersecting joints during multistep creep tests. Moreover, the creep fracture types are analysed. Finally, the existing damage characterization formula is improved based on the acoustic emission test parameters. The research findings provide a theoretical basis for the long-term stability assessment and monitoring of fissured rock masses and crack propagation trends in underground engineering projects.

## Methodology and experimental work

### Sample preparation

Based on on-site sampling and geological data, the sample material used was natural light yellow fine-grained feldspar sandstone (Yunnan, China), with a porosity of 10 ± 0.5% and a mineral composition of 40% quartz, 30% plagioclase feldspar, 10% calcite, 17% clay, 2% potassium feldspar and 1% anhydrite. After investigation, the stratigraphic structure in the study area is mainly composed of Upper Paleozoic and Mesozoic, and siltstone is the main lithology. The Upper Paleozoic strata are Paleozoic strata, mainly distributed in the southern part of the study area. Mesozoic strata are middle Paleozoic strata, mainly distributed in the northern part of the study area. Siltstone is a kind of sedimentary rock composed of fine grained sand and stone, which has high compactability and weathering resistance. Through laboratory analysis, the siltstone shows the following characteristics: fine particles, mainly composed of quartz sand and mica; Large block size and compact grain structure; Low porosity and poor water permeability; High compressive strength, suitable for building materials.

**Figure 3:**
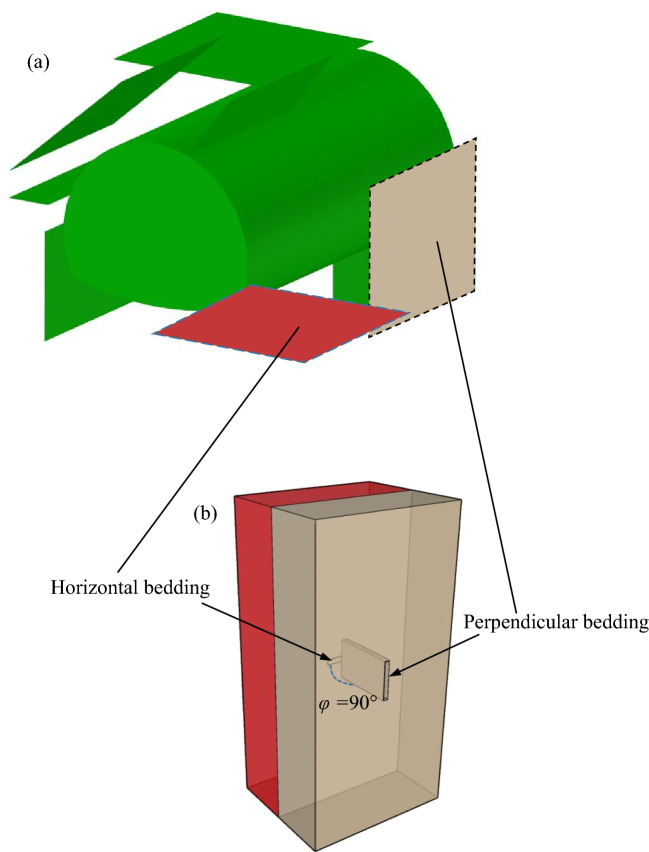
Schematic diagram of model setup: (**a**) tunnel with the two typical cross joints (**b**) simplified rock specimen with the two sets of cross-persistent joints.

Then, the rock collected on site was processed to obtain 60 × 25 × 100 mm cubic specimens, the rock mass joints were simulated by using a water jet system to cut 30-mm-long and 1-mm-wide slits in the specimens, 4 types of anisotropic joint samples were prepared by changing the joint inclination angle (*φ* = 0°, 30°, 60°, and 90°), and the P-wave velocity of the natural sandstone sample was measured to be approximately 1.97–2.12 km/s. In the tunnel excavated in the on-site rock mass shown in Fig. 3a, the surrounding rock on the right exhibited two typical cross joints, which were simplified to two 60 × 25 × 100 mm cube specimens bonded with epoxy resin glue for ease of indoor experimental investigation. These cross joints are shown in Fig. 3b. Red represents a specimen with a joint inclination angle *φ* = 0°, and green represents a specimen with a joint inclination angle *φ* = 60°, which are bonded together to form sandstone specimens with intersecting joints.

### Experimental methodology

**Figure 4:**
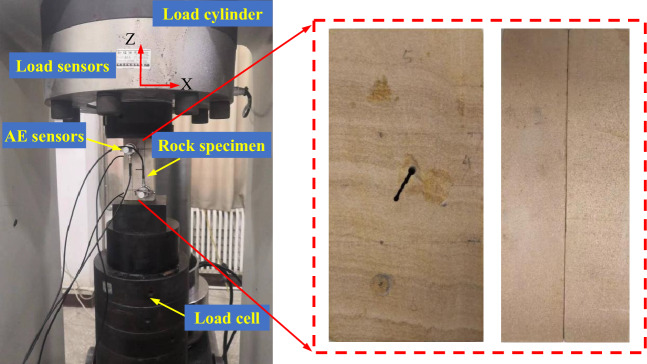
Servo-controlled rock creep testing machine.

Changes in temperature could influence the creep test results^22^. Thus, all the tests were performed at a constant room temperature of 26°. A servo-controlled TAW2000 triaxial creep testing machine was used to evaluate the joint specimens, as shown in Fig. 4. The maximum axial load of the testing machine was 2000 kN. The loading cylinder and the pressure sensor were at the top of the testing machine. Many layers of rigid cushion blocks were at the bottom of the testing machine. Moreover, the specimens were placed between the loading cylinder and the rigid cushion block. The final friction effect of the specimens influenced the test results^23^. Thus, the upper and lower ends of the specimen were coated with Vaseline. In all experiments, the axial strain was continuously measured using the LVDT displacement sensor, the pressure sensor on the top of the testing machine recorded the load on the specimen, the loading cylinder was servo-controlled to provide a stable load for a long time during rheological testing, and the maximum range of variation of the load was ± 5 N. Some scholars have carried out rock damage indoor tests by considering acoustic emission (AE) parameters^24,25^. We installed acoustic emission (AE) sensors on the front and rear surfaces of the specimen to monitor the evolution of crack damage throughout the process. To improve the positioning accuracy of the AE and to accurately calibrate the sound velocity, a lead break test was needed. The test showed that the threshold was required to be set below 5 mV to control environmental noise in acoustic emission acquisition.

Considering that the nonuniformity and anisotropy of the natural rock mass structure could produce different mechanical behaviours and affect the test results, to reduce the discreteness of test results under the same loading conditions, many of the specimens from the same batch of rock masses were subjected to repeated creep loading for long periods. In addition, possible midpoint obstacles, such as power failure and test equipment failure, took considerable time input from test personnel and test specimens. Thus, to overcome the above problems, we performed a multistep loading creep test on a specimen to obtain relatively accurate test results^26,27^. Before performing the creep test, a uniaxial compression test with a constant strain rate of 1.0 × 10^–5^ s^−1^ was carried out on the sandstone, and the short-term deformation and failure characteristics of the sandstone were obtained. As listed in Table 1, the uniaxial compressive strengths of the sandstone samples with intersecting joint were σc0°/0° = 33.93 MPa, σc0°/30° = 28.57 MPa, σc0°/60° = 26.88 MPa, and σc0°/90° = 22.16 MPa, respectively, and the uniaxial strengths of these samples could provide a reference for creep stress levels in the later stages.

**Table 1:** Physical and mechanical parameters of the different joint combination sandstone samples.

No	Rock type	Joint inclination (°)	Thickness (mm)	Width (mm)	Length (mm)	Uniaxial compressive strength (MPa)	Young’s modulus (GPa)
1		0	24.72	60.15	119.82	33.25	5.91
2		30	24.91	60.25	119.95	20.15	2.94
3		60	24.86	60.45	119.86	22.01	4.93
4		90	25	59.6	120.05	28.93	5.01
1–1		0/0	49.18	59.61	119.11	33.93	6.19
1–2		0/30	50.11	60.70	120.07	28.57	4.65
1–3		0/60	49.37	60.21	120.00	26.88	4.11
1–4		0/90	50.05	59.8	119.4	22.16	3.49

1–1 Joint inclination 0°/0° in red color, 1–2 Joint inclination 0°/30° in red and white color, 1–3 Joint inclination 0°/60° in red and green color, 1–4 Joint inclination 0°/90° in red and brown color.

Brace et al.^28^ and Heap et al.^29^ suggested that there was a crack damage starting point for rock deformation. Zhu et al.^30^ suggested that the creep load should be set to a value higher than 60% of the uniaxial compressive strength of the rock and that the rock below this value would not undergo creep damage. Therefore, we divided the stress levels of the multistep loading creep test into three phases: the first stage (11.67 MPa < 60% of the UCS), the second stage (25 MPa > 60% of the UCS), and the third stage (38.34 MPa≈100% of the UCS). At the beginning of the period, the rock specimen was loaded to the predetermined stress level of 11.67 MPa at a constant rate of 5 MPa/min. Then, the loading stress was held constant, and the rock specimen was allowed to deform for 18 min. After 18 min, the rock specimen was still loaded to the predetermined stress level of 25 MPa at a rate of 5 MPa/min. When the stress reached a high stress level of 25 MPa, a constant stress was maintained for 18 min, and the rock crept at this stage. After 18 min, the rock specimen was subjected to quasistatic loading at the same rate of 5 MPa/min, and the stress level was 38.34 MPa. The rock specimen underwent accelerated creep rupture at this stage, and some specimens did not reach a stress of 38.34 MPa and failed during quasistatic loading. The damage and rupture evolution of the rock could be reflected by measuring the axial strain, output AE energy, acoustic emission ring count, duration, rise time and amplitude values that were recorded for each test.

## Results

### Uniaxial compression test

**Figure 5:**
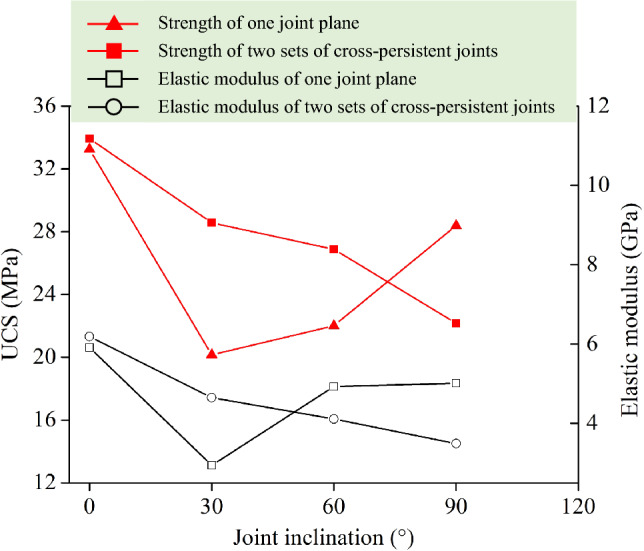
Quasi-static uniaxial compressive strength and elastic modulus of sandstone specimens under different joint inclinations.

To determine the stress level of the multistep creep test, we first carried out a uniaxial compression test on a sandstone sample with a single joint, as shown in Table 1. The angles of different prefabricated crack defects were 0°, 30°, 60°, and 90°. The sandstone specimen strengths of a single crack defect were *σ*_c0°_ = 33.25 MPa, *σ*_c30°_ = 20.15 MPa, *σ*_c60°_ = 22.01 MPa, and *σ*_c90°_ = 28.93 MPa, and the elastic moduli were *E*_0°_ = 5.91 GPa, *E*_30°_ = 2.94 GPa, *E*_60°_ = 4.93 GPa, and *E*_90°_ = 5.01 GPa. As shown in Fig. 5, with increasing joint inclination angle, the strength and elastic modulus values of the sandstone specimens with single joints first decreased rapidly and then increased slowly, and the whole strength and elastic modulus curve was U-shaped. This curve arose because when the prefabricated crack defect angle was 30°, the specimen was susceptible to shear‒slip failure, the peak strength was close to the minimum value, and the specimens at other angles still had a certain bearing capacity after the peak. The sandstone specimens with different intersecting joint angles of 0° and 0°, 0° and 30°, 0° and 60°, and 0° and 90° had strengths of σ_c0° and 0°_ = 33.93 MPa, σ_c0° and 30°_ = 28.57 MPa, σ_c0° and 60°_ = 26.88 MPa, and σ_c0° and 90°_ = 22.16 MPa, respectively, and elastic moduli of E_0° and 0°_ = 6.19 GPa, E_0° and 30°_ = 4.65 GPa, E_0° and 60°_ = 4.11 GPa, and E_0° and 90°_ = 3.49 GPa, respectively. According to the test results, with increasing angle of the intersecting joints, both the strength and elastic modulus of the intersecting joint specimen decreased. The prefabricated intersecting joint angle was 0°, and the joint gradually closed during the loading process. At this time, the specimen was similar to the intact rock; thus, the specimen had the highest strength. When the angle of the prefabricated intersecting joints was 30° or 60°, it was close to the shear failure angle of the nondefective sample. Specifically, the specimen body was susceptible to shear failure at this angle. When the angle of the prefabricated intersecting joint was 90°, some of the joint angles were parallel to the loading direction, resulting in tensile failure. The strength of shear failure was greater than that of tensile failure.

### Uniaxial multistep loading creep test with different joint angles

Due to the heterogeneity of natural rock, the indoor test results of rock specimens were highly discrete. Thus, in the uniaxial creep test performed on specimens with different joint angles, we used a multistep loading method, where different stresses were loaded on each specimen. Table 2 shows the results of the creep tests on the sandstone samples with intersecting joint angles of 0°/0°, 0°/30°, 0°/60°, and 0°/90° in three loading stages of stress, namely, 11.67 MPa (30% of the UCS), 25 MPa (65% of the UCS) and 38.34 MPa (100% of the UCS). By taking the 0°/30° specimen as an example, we determined that the strain increased by 0.435 × 10^–3^ in 18 min in the first creep stage and by 0.73 × 10^–3^ in 18 min in the second creep stage. Furthermore, the predetermined stress level was not reached in the third creep stage, and instantaneous failure occurred. The 0°/60° specimens had different results; the strain increased by 0.77 × 10^–3^ in 18 min in the first creep stage and by 0.54 × 10^–3^ in 18 min in the second creep stage. In addition, accelerated creep failure occurred after 14 min of the third creep stage. Apparently, the strain change of the 0°/30° specimen increased in the first two creep stages, while the strain change of the 0°/60° specimen decreased in the first two creep stages, indicating that the final failure mode could have occurred from the trend of the creep strain change.

**Table 2:** Results of creep tests for sandstone specimens with different joint inclinations.

Joint inclination (°)	Young's modulus (GPa)	Loading conditions	The first creep			The second creep			The third creep			
			The first stress level (MPa)	Strain increase (10^–3^)	Test duration (minute)	The second stress level (MPa)	Strain increase (10^–3^)	Test duration (minute)	The third stress level (MPa)	Strain increase (10^–3^)	Test duration (minute)	Failure type
0 & 0	4.85	Three-stage creep	11.67	0.379	18	25	1.354	18	32.84	11.845	0.73	Failure during load process
0 & 30	4.48	Three-stage creep	11.67	0.435	18	25	0.73	18	34.27	10.895	1.25	Failure during load process
0 & 60	3.79	Three-stage creep	11.67	0.77	18	25	0.54	18	38.34	10.645	14	Accelerating creep
0 & 90	5.54	Three-stage creep	11.67	0.25	18	25	0.729	18	33.98	11.25	1.97	Failure during load process

**Figure 6:**
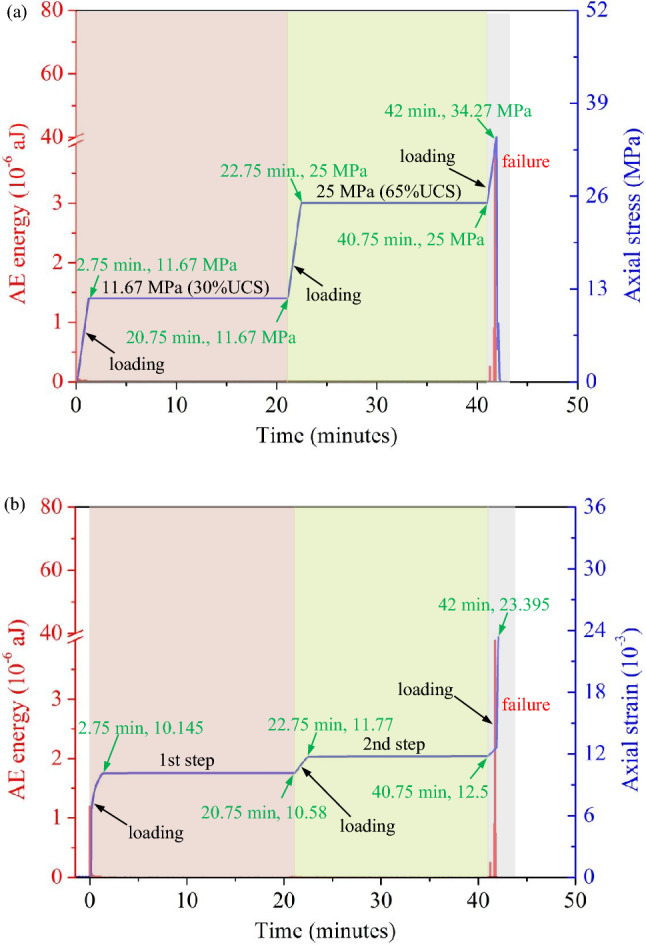
The curves for the AE released energy of the rock sample with the two sets of cross-persistent joints (0°/30°): (**a**) AE released energy vs. stress-time curve; (**b**) AE released energy vs. strain–time curve.

The axial stress, axial strain and AE energy curves of the sandstone specimens with intersecting joint angles of 0°/30° in the uniaxial creep test are shown in Fig. 6; the red bar graph corresponding to the left axis shows the degree of damage of the sandstone specimen under creep expressed by the AE energy. As shown in Fig. 6a, in the first step, the specimen was loaded to 11.67 MPa (40% of the UCS) and underwent creep for 18 min. As shown in Fig. 6b, during this period, the specimen strain increased from 10.145 × 10^–3^ to 10.58 × 10^–3^ (increased by 4.28%), and no obvious acoustic emission signal appeared, indicating that the rock did not undergo creep damage under the condition of 11.67 Mpa (< 60% of the UCS) in the first step. As shown in Fig. 6a, during the second step, the specimen was loaded to 25 MPa (65% of the UCS) and underwent creep for 18 min. As shown in Fig. 6b, during this period, the specimen strain increased from 11.77 × 10^–3^ to 12.5 × 10^–3^ (increased by 6.2%), followed by a third loading. Significant acoustic emission occurred during loading, and the specimen underwent instantaneous failure at 42 min. At this time, the strain peaked at 23.395 × 10^–3^, and the acoustic emission energy peaked at 3.95 × 10^–6^ aJ.

**Figure 7:**
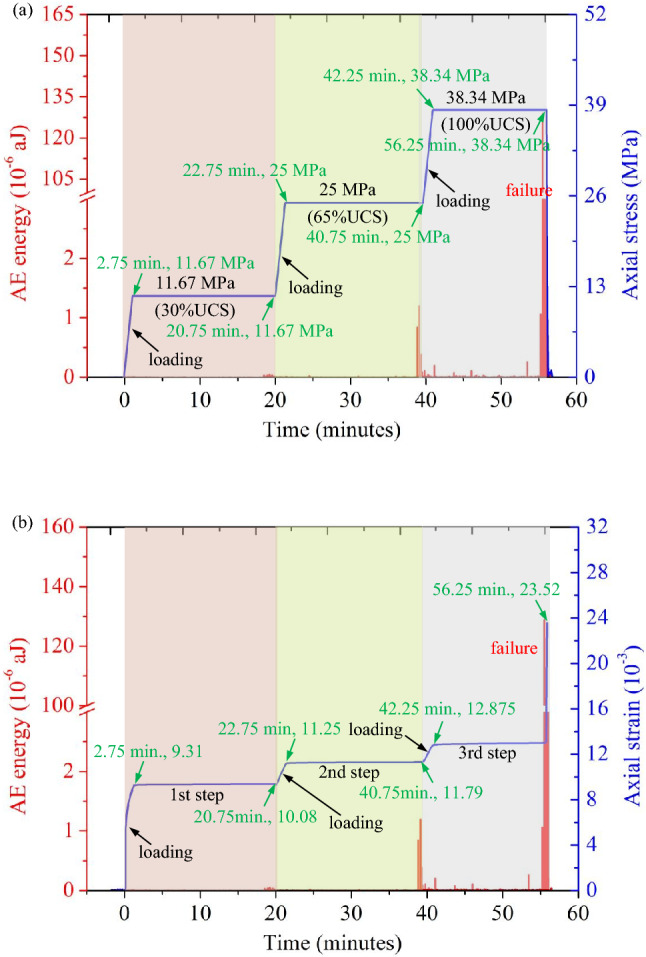
The curves for the AE released energy of the rock sample with the two sets of cross-persistent joints (0°/60°): (**a**) AE released energy vs. stress-time curve; (**b**) AE released energy vs. strain–time curve.

Figure 7 shows the axial stress, axial strain and acoustic emission (AE) energy curves of a 0°/60° combined sandstone specimen under uniaxial creep. As shown in Fig. 7a, in the first step, the specimen was loaded to 11.67 MPa (43.9% of the UCS) and underwent creep for 18 min. The corresponding strain increased from 9.31 × 10^–3^ to 10.08 × 10^–3^ (increased by 8.27%), as shown in Fig. 7b. At this stage, no significant acoustic emission signal appeared. In the second step, as shown in Fig. 7a, the specimen was loaded to 25 MPa (93% of the UCS) and underwent creep for 18 min under a constant stress, and the corresponding strain increased from 11.25 × 10^–3^ to 11.79 × 10^–3^ (increased by 4.8%), as shown in Fig. 7b. The third loading process started at *t* = 40.75 min, as shown in Fig. 7a. When the loading stress reached 38.34 MPa, the stress at this time was already greater than 100% of the UCS of 26.88 MPa, followed by creep. When *t* = 56.25 min, Fig. 7a and b show that the stress decreased sharply from 38.34 MPa, and the strain increase changed from the original slow increase to a rapid increase. At this time, the specimen lost its continued bearing capacity, the strain reached a maximum value of 23.52 × 10^–3^, and the specimen underwent accelerated creep failure. According to the above experimental results, when the specimen was loaded to a stress level greater than the uniaxial strength UCS, the specimen could still bear the load, indicating that the current rock engineering design was conservative for the uniaxial strength UCS. Furthermore, the engineering bearing capacity could be determined according to the long-term strength of rock. Figure 7b shows that the acoustic emission energy was continuously output in the third creep stage, and the acoustic emission energy reached a maximum value of 130 × 10^–6^ aJ, indicating that creep damage continued to occur in the specimen.

### Spatiotemporal evolution of typical AE acoustic emissions

**Figure 8:**
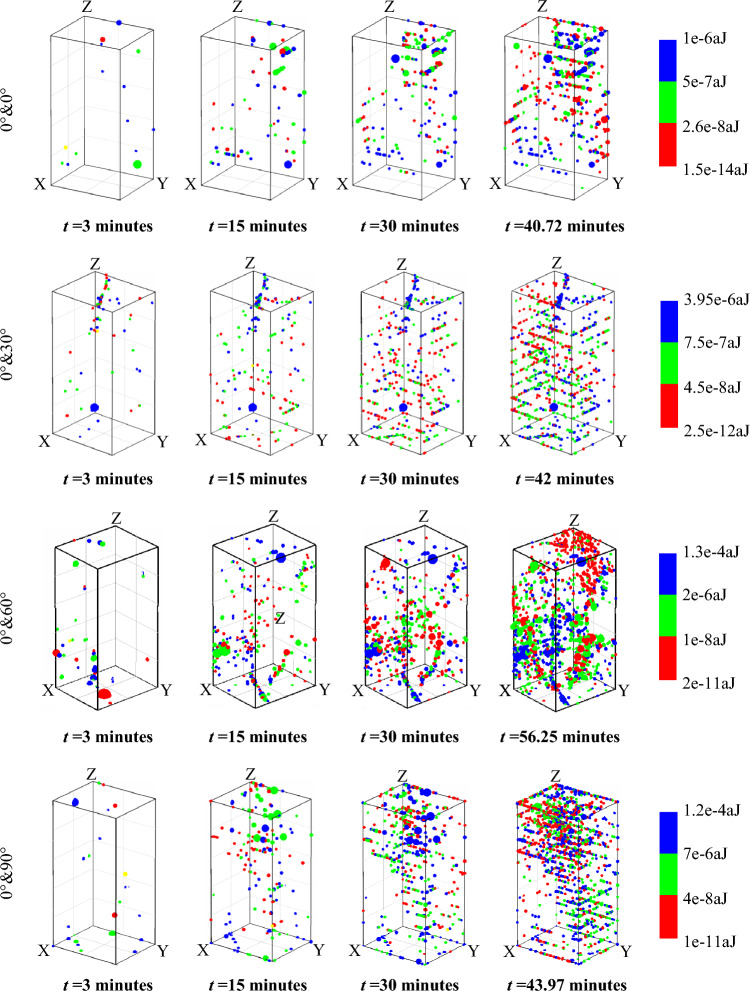
The time histories of AE localization of the creep rock sample with the two sets of cross-persistent joints.

To a certain extent, the spatiotemporal distribution of AE acoustic emission could reflect the damage evolution of the rock creep process^31^, and Fig. 8 shows the three-dimensional spatial positioning results of the AE events of sandstone samples with intersecting joint angles of 0°/0°, 0°/30°, 0°/60°, and 0°/90° under the same three-stage load. A small number of AE events were sparsely distributed in the sandstone samples after the initial loading time of *t* = 3 min, indicating that the 0°/0° ~ 90° combined intersecting joint initially had little effect on the unstable growth of cracks under low stresses. However, there were significant differences in the distributions of AE events at the end of the first creep stage at *t* = 15 min for specimens with different intersecting joint angles, and there were few AE events for the 0°/0° specimens. The AE events of the positioned 0°/60° specimens were clustered in the lower left corner, and the AE events of the positioned 0°/90° specimens were clustered in the upper right corner, indicating that stress concentration occurred in these regions. At the end of the second creep stage, *t* = 30 min, as the creep loading level increased, the AE events of the 0°/60° specimens were highly clustered, forming an X-shaped region. The AE events of the 0°/90° specimens were approximately clustered in the upper right corner, forming a distinct shear plane. Compared with the axial stress, axial strain and acoustic emission (AE) energy curves in Fig. 7, the spatiotemporal evolution of AE acoustic emission in Fig. 8 could reflect the internal damage characteristics of the first and second stages of creep, although the AE energy and axial strain values reflected in the first and second stages of creep in Fig. 7 were poor. The AE events of the 0°/0° and 0°/30° specimens were relatively uniform and sparse, indicating that the internal damage was uniformly distributed during this period. In the third creep stage, the 0°/0°, 0°/30°, and 0°/90° sandstone specimens were not loaded to the predetermined value of 38.34 MPa and failed; in particular, the 0°/90° sandstone specimens had shear surfaces obviously composed of AE events, indicating that the shear stress was clustered in this direction. Nevertheless, the 0°/0° and 0°/30° AE events were relatively uniformly and sparsely distributed, indicating that there was no stress concentration. The 0°/60° specimen underwent accelerated creep at *t* = 56.25 min. Finally, an X-shaped tensile‒shear mixed fracture zone consisting of AE events was formed. Based on the above results, when the intersection angles of the prefabricated joints of the specimen were small or parallel, the two prefabricated joints had little influence on each other during the creep process, and the final rupture process was very mild. However, when the intersection angle of the prefabricated joints of the specimen was large, unpredictable accelerated creep failure was very likely to occur, and the resulting rupture process was violent.

### Rupture mode

**Figure 9:**
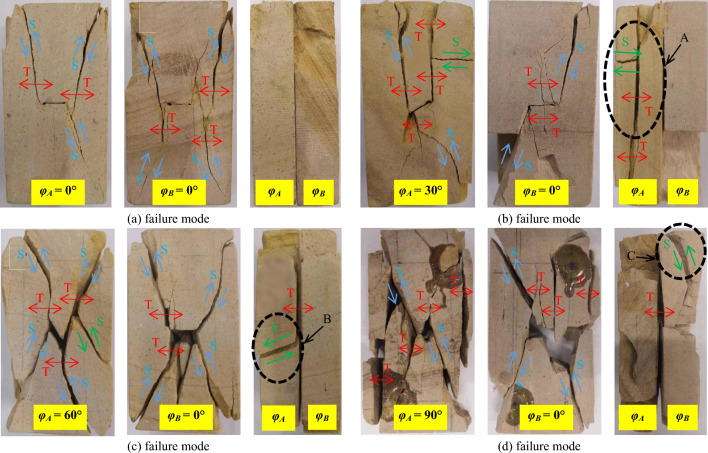
Rupture modes of sandstone specimens with the two sets of cross-persistent joints for different angles: (**a**) *φ*_*A*_ = 0°, *φ*_*B*_ = 0°; (**b**) *φ*_*A*_ = 30°, *φ*_*B*_ = 0°; (**c**) *φ*_*A*_ = 60°, *φ*_*B*_ = 0°; (**d**) *φ*_*A*_ = 90°, *φ*_*B*_ = 0°.

The crack propagation modes of the rock samples could be divided into 3 categories: tensile cracks (type I), shear cracks (type II) and mixed tear cracks (type III). Figure 9 shows the macroscopic failure modes of sandstone samples with different intersecting joint angles of 0°/0°, 0°/30°, 0°/60°, and 0°/90°, where T and S represent tensile cracks and shear cracks, respectively. According to Fig. 9a, the failure cracks of the two specimens of the 0° and 0° sandstone specimens were similar, the specimen was compressed at the beginning, a stress concentration was generated along both ends of the 0° prefabricated crack, and a typical upwards type I tension crack T gradually appeared along the loading direction. In the subsequent third loading stage, due to the lateral expansion of the specimen, type II shear crack S further expanded and developed into a U-shaped crack. In addition, the acoustic emission positions at 0°/0° in Fig. 8 also indicate that crack propagation mainly occurred in the upper part of the specimen. The specimens observed from the side (Y-axis direction) did not separate, indicating that two identical prefabricated joints of 0°/0° had little effect on each other during loading. Figure 9b shows two specimens of the 0°/30° sandstone specimen after creep loading and failure. The *φ*_*A*_ = 30° specimen first underwent a type I tension crack T near the prefabricated crack because the tensile strength of the rock was much lower than the compressive strength, and the stress concentration around the prefabricated cracks created a tensile region^32^. Under the loading action, the stress concentration of the original crack tip gradually shifted toward the upper and lower ends of the specimen with the propagation of the tension crack T. In contrast, the *φ*_*B*_ = 0° specimen deformed and expanded in the X-axis direction under the action of loading in the third stage so that the stress acting on the parallel loading direction at both ends of the 0° prefabricated crack was gradually deflected, and the type I tension crack T gradually transformed to the type II shear crack S. Notably, area A of the specimen was partially peeled off due to the vertical tension crack T in the side view (Y-axis direction), which indirectly caused the type III mixed tear crack to generate a shear stress crack (the green arrow in Fig. 9b).

Figure 9c shows the morphologies of two specimens of the 0°/60° sandstone specimen after creep loading and failure. The figure shows that type I tension crack T appeared near the prefabricated crack of the two specimens *φ*_*A*_ = 60° and *φ*_*B*_ = 0° because as the load increased, the tensile stress near the prefabricated crack increased^34^. When the tensile strength was exceeded, cracking parallel to the loading direction occurred at the stress concentration point near the prefabricated crack. When the type I tension crack T expanded to a certain length, the stress concentration point shifted away from the tip point of the prefabricated crack, and the creep deformation and expansion of the specimen caused the crack to further propagate. However, at this time, the stress direction was deflected due to the expansion deformation of the specimen, the prefabricated crack direction of the specimen *φ*_*A*_ = 60° was close to the shear failure angle of the nondefective specimen, and the specimen crack further expanded at this angle, eventually accelerating creep failure to form X shear cracks. The *φ*_*B*_ = 0° prefabricated crack specimens were influenced by the failure of the *φ*_*A*_ = 60° specimens, and eventually, X-shear cracks also formed, indicating that the shear stress generated by the *φ*_*A*_ = 60° specimens acted on the *φ*_*B*_ = 0° specimens. As shown in Fig. 9c, the type III mixed tear crack B area appeared on the sides of the *φ*_*A*_ = 60° and *φ*_*B*_ = 0° specimens, which could be attributed to the resultant force of the shear stress that produced X-shear cracks on the *φ*_*A*_ = 60° specimen; the reaction stress on the *φ*_*A*_ = 60° specimen was generated by the compressive expansion of the specimen *φ*_*B*_ = 0° (indicated by the green arrow in area B).

As shown in Fig. 9d, the two 0°/90° sandstone specimens had similar failure modes after creep loading and failure. The failure modes were apparent along the shear failure in the oblique direction (approximately 45°) from the upper left to the lower right, which was similar to the changes in the AE events of the 0°/90° sandstone specimens shown in Fig. 8. A vertical type I tension crack T appeared near prefabricated cracks with *φ*_*A*_ = 90° and *φ*_*B*_ = 0° because tensile stress occurred in these sites at the beginning of loading. When the loading stress increased, the type I tensile cracks and T cracks gradually grew under the action of long-term tensile stress at these sites. Notably, *φ*_*A*_ = 90° did not eventually undergo vertical cracking in the parallel loading direction, indicating that the tensile stress of *φ*_*A*_ = 90° was influenced by *φ*_*B*_ = 0° deformation. Specifically, the resultant force direction of *φ*_*A*_ = 90° horizontal tensile stress and *φ*_*B*_ = 0° vertical compressive stress was 45°, where the shear cracks were very likely to grow. The sides of the two specimens, *φ*_*A*_ = 90° and *φ*_*B*_ = 0°, were also type III. The mixed tear crack C area bonded together before loading and separated after the two specimens failed.

## Discussion

### Quantification of creep cracks according to the rise angle (RA) and average frequency (AF) methods

**Figure 10:**
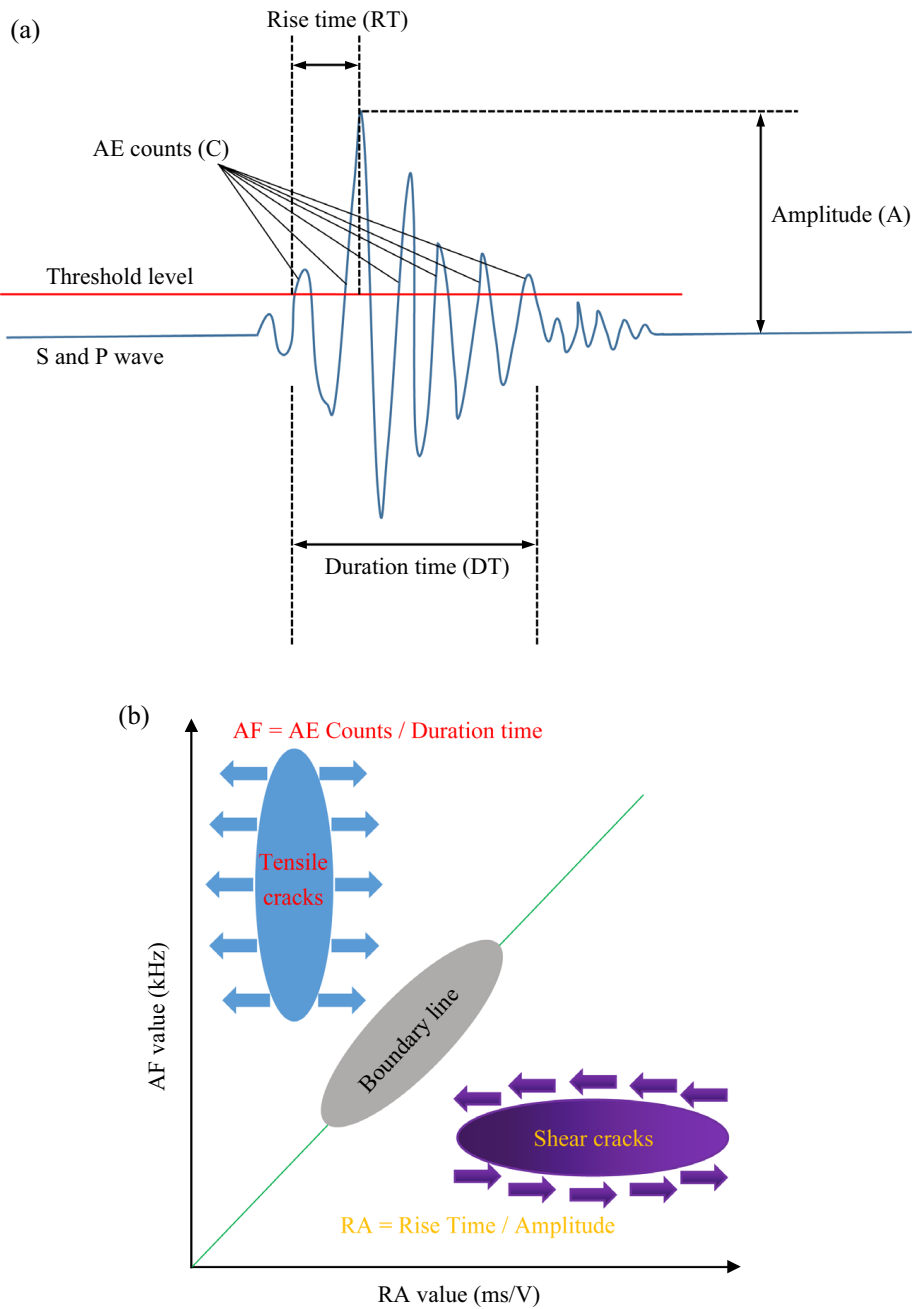
AE parameters characterization and tensile-shear crack classification method: (**a**) AE waveform associated with rock fracture; (**b**) typical fracture modes based on RA and AF.

According to the results of the creep loading test, crack growth occurred in jointed rocks, and in Reference^33^, image technology was employed to identify the degree of rupture under creep loading in single-jointed sandstone. In Reference^34^, loading and unloading cycle creep tests were performed on sandstone, and the axial strain rate and lateral strain rate were used to represent the degree of rock crack evolution. The above results only qualitatively accounted for the tensile cracks and shear cracks in rock failure during the creep process and did not quantitatively explain the effects of tensile cracks and shear cracks on rock creep failure. Studies showed that the RA–AF value in acoustic emission could be used to quantitatively characterize crack types in material structures^35^. When the jointed rock underwent deformation under the pressure load, tensile cracks formed at the site where the joint stress was concentrated. Strain energy was released in the form of a stress wave^36^, which was received by AE sensors. As shown in Fig. 10a, typical rock fracture resulted in a stress waveform, with blue curves representing the P and S stress waves and red representing the threshold value set for AE noise filtering to receive a valid signal. An acoustic emission stress wave had multiple wave peaks. When multiple peaks exceeded the set threshold, these peaks were counted as ringing AE counts (C), and the time interval elapsed between the first peak crossing the threshold and the last peak decreasing to the threshold was called the duration time (DT) (in μs), where the average frequency AF in acoustic emission was as follows:

1 $${\text{AF}} = {\text{Counts}}/{\text{Duration}},\;{\text{kHz}}$$

As shown in Fig. 10a, the stress waveform occurred under typical rock fracture. There were also two important parameters. One important parameter was the rise time (RT) in μs, which represents the time elapsed from the waveform initially crossing the threshold to the maximum amplitude. The other important parameter was the maximum amplitude (A) in mv of the waveform, where the rising angle RA in the acoustic emission was as follows:

2 $${\text{RA}} = {\text{Rise}}\;{\text{Time}}/{\text{Amplitude}},{\text{ms}}/{\text{V}}$$

As shown in Fig. 10b, rock specimens primarily underwent two modes: tensile failure and shear failure under the action of load. The X-axis represents the RA value, the Y-axis represents the AF value, and the green line in the middle represents the classification boundary of tensile failure and shear failure. If the values of the points (RA, AF) fell within the upper part of the dividing line, the rock had a tensile crack, and if the values of the points (RA, AF) fell within the lower part of the dividing line, the rock had a shear crack. Specifically, the tensile mode corresponded to a high amplitude and had a short rise time and duration time, resulting in a low RA and high AF. In contrast, the shear mode showed a relatively low amplitude, with a long rise time and duration time, resulting in relatively high RA values and low AF values^37,38^.

**Figure 11:**
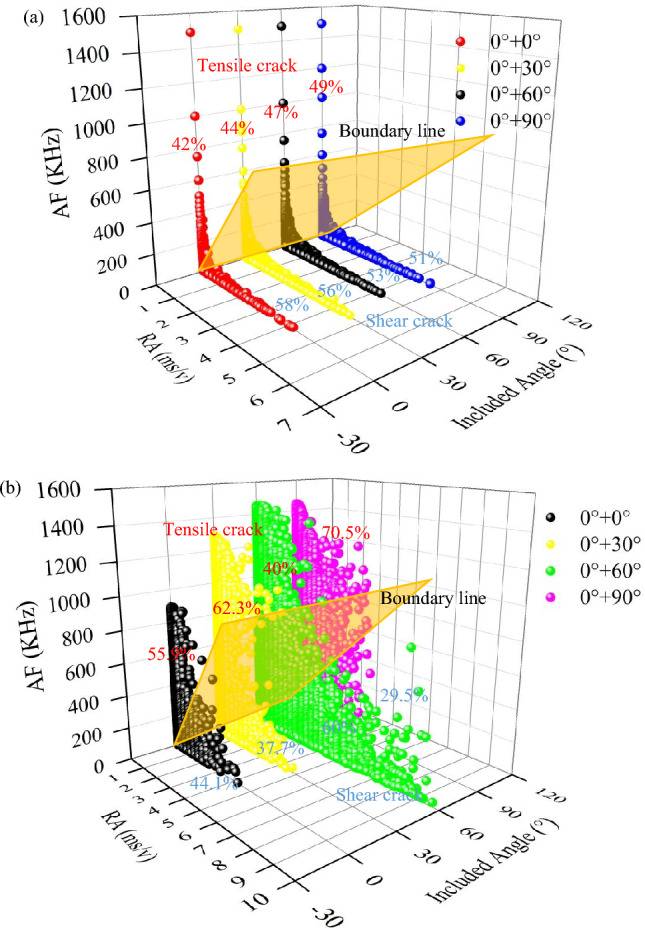
Variation in AF vs RA values with the two sets of cross-persistent joints for different angles: (**a**) uniaxial compressive tests; (**b**) uniaxial creep tests.

To analyse and study the influences and mechanisms of sandstone samples with different intersecting joint angles of 0°/0°, 0°/30°, 0°/60°, and 0°/90° on the propagation of cracks, we analysed the fracture modes of different intersecting joint specimens in the uniaxial compression test and uniaxial creep test from two perspectives: rising angle (RA) and average frequency (AF). As shown in Fig. 11a, different intersecting joints of the 0°/0°, 0°/30°, 0°/60°, and 0°/90° specimens had uniform distribution ranges of AF values and RA values under the same uniaxial loading conditions. The maximum AF value was 1500 kHz, and the maximum RA value was 4.5 ms/V. This finding indicated that even if different intersecting joints were combined, little differences arose among the final damage degrees of the specimens. The specimens were arranged in ascending order of intersecting joint size after complete damage. The tensile events accounted for 42%, 44%, 47% and 49% of the total events, and the shear events accounted for 58%, 56%, 53% and 51% of the total events for the 0°/0°, 0°/30°, 0°/60°, and 0°/90° specimens, respectively. There was little difference between the numbers of shear events and tensile events. As a result, many specimens underwent macroscopic tensile shear failure after uniaxial loading^39^. After the creep test, as shown in Fig. 11b, the 0°/0°, 0°/30°, and 0°/90° intersecting joint specimens (RA, AF) had additional tensile crack zones; the range of RA was 0 ~ 5 ms/V, and the range of AF was 0 ~ 1500 kHz. With increasing intersecting joint angle, the acoustic emission signal points occupied 55.9%, 62.3% and 70.5% of the tensile crack zones for the 0°/0°, 0°/30°, and 0°/90° specimens, respectively. Thus, the final failure modes of the 0°/0°, 0°/30°, and 0°/90° intersecting joint specimens were dominated by tensile failure after the multistep creep stage. According to Fig. 9a, many tensile cracks formed near the horizontally prefabricated joints. As shown in Fig. 9b, the *φ*_*A*_ = 30° specimen had tensile cracks near the prefabricated joint that eventually propagated to the top under the action of long-term prolonged loading. The *φ*_*A*_ = 30° specimen underwent apparent tensile cracking from top to bottom on its sides. According to Fig. 9d, the 0° and 90° intersecting joint specimen had many tensile cracks on the surface, and the middle crack gaps of the side specimen were large. The RA and AF distributions in Fig. 11b also show that the RA value distribution of the 0°/60° intersecting joint specimen was wide. Moreover, the RA range was 0 ~ 10 ms/V, which was approximately two times greater than the RA value distribution ranges of the other specimens. This finding indicated that the 60-degree specimen had additional shear cracks and that the specimens at other bedding angles had additional tensile cracks.

### Improved damage law based on acoustic emission parameters

According to the AE energy–time and strain‒time curves shown in Figs. 6 and 7, different intersecting joints in the multistage creep process were found to have nonnegligible effects on the damage and deformation characteristics of the specimen. The 0°/0°, 0°/30°, and 0°/90° intersecting joint specimens finally underwent tensile failure under instantaneous loading, while the 0°/60° specimens finally underwent hysteresis-accelerated creep failure. To quantify these damage trends, many scholars have made enormous efforts in recent years. Zhu et al.^39^ believed that the rock would develop maximum compressive principal strain once damaged. The specific expression is provided in Eq. (3). Here, the range of *D* was 0 ~ 1, and the larger the value was, the more severe the damage.

3 $$D = 1{ - }\left| {\frac{{\varepsilon^{\prime}}}{\varepsilon }} \right|^{2}$$

**Figure 12:**
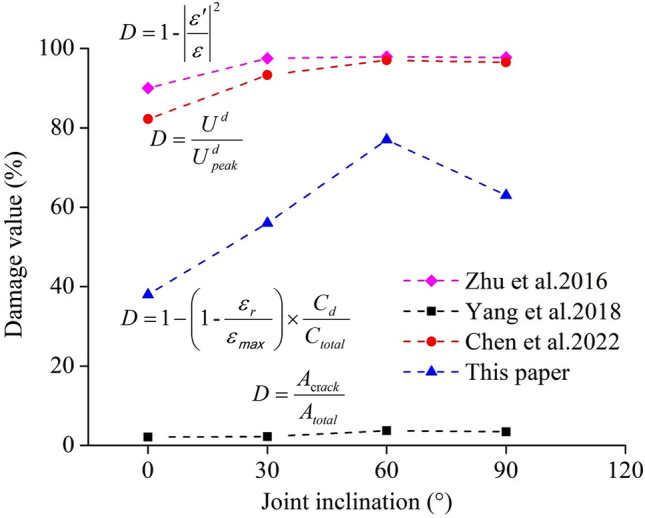
Relationship between the modified damage variable *D* and the joint inclinations.

We substituted the maximum compression principal strain data of the creep tests performed on specimens with different intersecting joints—0°/0°, 0°/30°, 0°/60°, and 0°/90°—into Eq. (3) to obtain the damage trends. As shown by the points marked by pink diamonds in Fig. 12, the *D* gradually increased from 90% to 97.9% with increasing intersecting joint angle. It was clear that an overly high value was unreasonable. Yang et al.^40^ defined the damage variable by calculating the ratio of the area damaged after rock loading to the area not damaged before loading, as shown in Eq. (4):

4 $$D = \frac{{A_{{{\text{cr}}ack}} }}{{A_{total} }}$$

where *A*_crack_ and *A*_total_ are the area of the microcracks and the total area of the specimens, respectively. Equation (4)was used to calculate the rupture area after specimens with different intersecting joints underwent creep, as shown in Fig. 9. As shown by the black square points and dashed lines in Fig. 12, the derived *D* value increased from 2.11% to 3.72% with increasing intersecting joint angle, and the calculated damage *D* value was small. This result arose because the secondary cracks and fine shear cracks in the image could not be identified, and this crack damage accounted for a large proportion of the total damage, indicating that the method of identifying damage using images should be improved.

According to the law of conservation of energy, rock loading is the process of storing energy, and rock damage and failure are the processes of releasing energy. Some scholars believe that damage or crack propagation during the rock loading process occurs in the form of dissipative energy^41^. Wang et al.^42^ fitted the experimental data by establishing a relationship between the rock damage variables and the variations in the dissipative energy of cyclic loading and unloading. Chen et al.^43^ defined the rock damage variable *D* by the dissipation energy at any time in the rock loading process and the dissipative energy corresponding to the peak intensity of the rock, as shown in Eq. (5):

5 $$D = \frac{{U^{d} }}{{U_{peak}^{d} }}$$

The dissipative energy data derived from the creep tests of specimens with different intersecting joint angles of 0°/0°, 0°/30°, 0°/60°, and 0°/90° were substituted into Eq. 5 to calculate the *D* value. As shown by the red circular points and dashed lines in Fig. 12, with increasing intersecting joint angle, *D* gradually increased, and the value range was 82.2% ~ 96.5%. Compared with the rupture mode at the end of creep in different intersecting joint specimens in Fig. 9, the *D* value was greater. In fact, rock damage mostly occurred internally, and acoustic emission monitoring was an effective method for detecting the occurrence of microcracks in rock samples under stress^44^. Zhao et al.^45^ introduced the characteristic parameter of the acoustic emission-ringing cumulative count into the expression of damage variables. Due to the creep test on different intersecting joints, the specimen stress was constant, and the strain gradually increased over time. Thus, we improved the damage variable in Reference^45^, as shown in Eq. (6):

6 $$D = 1 - \left( {1{ - }\frac{{\varepsilon_{r} }}{{\varepsilon_{{}} }}} \right) \times \frac{{C_{d} }}{{C_{total} }}$$

where *ε*_*r*_ is the residual deformation of the specimen after creep, *ε*_*max*_ is the peak strain of the specimen, *C*_*d*_ is the cumulative count of the acoustic emission ringing at a certain moment, and *C*_*total*_ is the cumulative count of the acoustic emission ringing throughout the process of creep. Equation (6) was used to calculate the post-creep damage *D* values of specimens with different intersecting joint angles of 0°/0°, 0°/30°, 0°/60°, and 0°/90°, which were 38%, 56%, 77% and 63%, respectively, as shown by the blue triangular mark points-dashed lines in Fig. 12. Due to accelerated creep failure at the final stage of 0°/60° creep loading, as shown in Fig. 9c, both *φ*_*A*_ = 60° and *φ*_*B*_ = 0° developed X-type shear cracks. No oblique cracks (approximately 45°) formed in the specimen after multistep creep^46^. This finding indicated that the intersection of the joints of the specimens inhibited and influenced the crack growth of any single prefabricated joint. A larger joint intersection angle led to a stronger impact. When *φ*_*A*_ = 60°, the joints tended to shear at the joint angle, but when *φ*_*B*_ = 0°, joint inhibition caused the corresponding inhibitory stress to occur in the normal direction at a joint angle of *φ*_*A*_ = 60°; thus, the joint stress concentration point propagated in one additional direction under the action of the inhibition stress. When a type III mixed tear crack appeared on the side of the specimen under creep loading, the crack propagation stress aggravated the crack propagation in this direction, and finally, the specimen became increasingly fragmented. Thus, the calculated *D* value was the largest. The accelerated creep failure of rock in engineering was difficult to predict, but a combination of rock joint angles could be used to predict hazardous areas prone to accelerated creep failure in advance, which could play a role in reducing engineering risks.

Based on the visualization results, when two different intersecting joints were combined (0°/30°, 0°/60°, and 0°/90°), type III mixed tear cracks occurred, and the 0°/60° specimens were susceptible to accelerated creep failure and finally to X-type fracture. However, the other specimens were susceptible to either instantaneous splitting failure or instantaneous shear failure. This result arose because the 60-degree specimens tended to undergo shear, leading to additional shear cracks. It took time for the shear stress to reach a high value, but the tensile stress quickly reached its strength. Finally, the 60-degree specimen tended to undergo hysteresis accelerated creep failure. The analysis showed that the effect of fracture on the specimens was affected mainly by the spatial relationship between the intersecting joints and the principal stress. The stress states parallel and perpendicular to the joint could directly affect the shear and tensile strengths of the joint and the relative sliding of different rocks along the joint, respectively. However, the stresses parallel and perpendicular to the joint had opposite effects on the differential deformation and interface damage and sliding characteristics.

Under the intersecting joints (0°/30°, 0°/60°, and 0°/90°), the specimens exhibited strong instantaneous rockburst tendencies, and the 0°/60° specimens exhibited time-delayed rockburst tendencies. Creep accelerated as the critical level of damage was reached, causing the 0°/60° specimen to fail. This result could explain why rock failure instability always occurred at some time when there was no external influence that could account for changes in the stress distribution. Thus, the two rockburst types were attributed to the accumulation of creep cracks and damage in rock specimens under a combined contribution of the creep stress state and inclination angle.

## Conclusions

In this study, uniaxial compression and multistep stress creep tests were conducted on sandstone specimens with different intersecting joint angles *φ* (joint angles *φ* = 0°, 30°, 60°, 90°). Acoustic emission (AE) parameters were used to characterize the spatiotemporal damage evolution characteristics of the time-dependent strain and fissure propagation of sandstone specimens. Moreover, these parameters were used to quantify the type I–II–III mixed failure damage values of sandstone specimens with intersecting joints (joint angles *φ* = 0°, 30°, 60°, 90°).

1. The uniaxial compressive strength and elastic modulus values of the sandstone specimens with a single crack defect at prefabricated crack angles of 0°, 30°, 60°, and 90° first decreased and then increased, exhibiting a U-shape with increasing prefabricated crack angle. Different intersecting joints changed this trend, and the uniaxial compressive strength and elastic modulus values of sandstone specimens with different intersecting joint angles of 0°/0°, 0°/30°, 0°/60°, and 0°/90° gradually decreased with increasing prefabricated crack angle.
2. After the multistep creep load (initial stage of 11.67 MPa (< 60% of the UCS), intermediate stage of 25 MPa (> 60% of the UCS)), the specimens with a joint angle of φ = 60° underwent accelerated creep failure under a third creep load of 38.34 MPa (> 100% of the UCS) of 26.88 MPa for 14 min, and the rupture of the *φ* = 60° rock specimens exhibited an X-shaped shear mode. The specimens with joint angles of *φ* = 0°, 30°, and 90° all underwent instantaneous failure during the third loading process, and the two specimens with joint angles of *φ* = 0° both underwent a splitting tensile failure mode. For the specimens with joint angles of *φ* = 30°, one specimen underwent a split tension failure mode (*φ*_*A*_ = 30°), and the other specimens underwent an oblique shear failure mode (*φ*_*B*_ = 0°). All specimens with joint angles of *φ* = 90° underwent an oblique shear failure mode. After failure, the specimens with joint angles of *φ* = 30°, 60°, and 90° had type I tension cracks T, type II shear cracks S and type III mixed tear cracks.
3. Acoustic emission technology could adequately characterize the microscopic damage evolution of sandstone during creep. The temporal evolution of the AE events coincided well with the axial creep curve, and the specimen with a joint angle of *φ* = 60° underwent accelerated creep failure in the third creep stage. Finally, the AE events formed an X-type tensile‒shear mixed rupture zone, and the acoustic emission energy peaked at 130 × 10^–6^ aJ. The prefabricated joints had small intersection angles or were parallel, had little mutual influence during creep, and eventually ruptured very mildly. However, when the intersection angle of the prefabricated joints of the specimen was large, unpredictable accelerated creep failure was very likely to occur, and the resulting rupture was violent.
4. The RA–AF method could effectively characterize the damage failure modes of sandstone specimens with intersecting joint angles of 0°/0°, 0°/30°, 0°/60°, and 0°/90°, and the distributions of the AF and RA values were basically equal under the same uniaxial loading conditions, indicating that the failure modes of the sandstone specimens were dominated by tensile‒shear failure. After the multistep creep test, with increasing intersecting joint angle, the 0°/0°, 0°/30°, and 0°/90° sandstone specimens exhibited acoustic emission signal points that occupied 55.9%, 62.3% and 70.5% of the tensile crack zone, respectively. The final failure modes of the intersecting joint specimens were dominated by tensile failure. Since the waveform shape parameter RA of the 0°/60° sandstone specimen remained high, the average frequency of the AF parameter was low, and finally, X-type shear fracture occurred.

## Summary

There are various joints in natural rock masses, which develop and activate during underground engineering construction due to stress disturbance and long-term ground stress. The development of joints can lead to various disasters, such as rockbursts. To date, however, most scholars have performed detailed research on type I–II cracks. The study of type III cracks is limited by experimental conditions. Herein, we determine the I–II–III mixed failure damage evolution characteristics of the time-dependent strain and fissure propagation of sandstone specimens with intersecting joints (joint angles φ = 0°, 30°, 60°, 90°). Different intersection joints affect and constrain each other during the force process, and eventually, different macroscopic ruptures occur. The specimens with joint angles φ = 0°, 30°, and 90° all undergo instantaneous failure during the third loading cycle. However, the specimen with a joint angle φ = 60° undergoes accelerated creep failure. The research results have a certain reference value for the early warning and risk mitigation of instantaneous and delayed rockbursts.

## Data Availability

The datasets used and/or analysed during the current study are available from the corresponding author upon reasonable request.

## References

[1] Sendir, H. & Yilmaz, I. Structural, geomorphological and geomechanical aspects of the Koyulhisar landslides in the North Anatolian Fault Zone (Sivas, Turkey). *Environ. Geol.* **42**, 52–60 (2002).

[2] Manouchehrian, A. & Cai, M. Analysis of rockburst in tunnels subjected to static and dynamic loads. *J. Rock Mech. Geotech. Eng.* **9**(6), 1031–1040 (2017).

[3] Barton, N., Bandis, S. C. & Bakhtar, K. Strength, deformation and conductivity coupling of rock joints. *Int. J. Rock Mech. Min. Sci. Geomech.* **22**(3), 121–140 (1985).

[4] Reik, G. A. & Currie, J. B. A study of relations between rock fabric and joints in sandstone. *Can. J. Earth Sci.* **11**(9), 1253–1268 (1974).

[5] Wang, M. Z. & Cai, M. Modeling of time-dependent deformation of jointed rock mass. *Rock Mech. Rock Eng.* **55**(4), 2049–2070 (2022).

[6] Li, D. Y., Xiao, P., Han, Z. Y., Zhu, Q. Q. & Wang, S. Y. Mechanical and failure properties of rocks with a cavity under coupled static and dynamic loads. *Eng. Fract. Mech.* **225**, 106195 (2020).

[7] Chang, S. H., Lee, C. I. & Jeon, S. Measurement of rock fracture toughness under modes I and lI and mixed-mode conditions by using disc-type specimens. *Eng. Geol.* **66**(1–2), 79–97 (2002).

[8] Ayatollahi, M. R. & Akbardoost, J. Size and geometry effects on rock fracture toughness: Mode I fracture. *Rock Mech. Rock Eng.* **47**, 677–687 (2014).

[9] Omidimanesh, M., Sarfarazi, V., Babanouri, N. & Rezaei, A. Investigation of fracture toughness of shotcrete using semi-circular bend test and notched Brazilian disc test, experimental test and numerical Approach. *J. Min. Environ.* **14**(1), 233–242 (2023).

[10] Bahrami, B., Ghouli, S., Nejati, M., Ayatollahi, M. R. & Driesner, T. Size effect in true mode II fracturing of rocks: Theory and experiment. *Eur. J. Mech.-A/Solids.* **94**, 104593 (2022).

[11] Bahrami, B., Nejati, M., Ayatollahi, M. R. & Driesner, T. Theory and experiment on true mode II fracturing of rocks. *Eng. Fract. Mech.* **240**, 107314 (2020).

[12] Aliha, M. R. M. & Ayatollahi, M. R. Rock fracture toughness study using cracked chevron notched Brazilian disc specimen under pure modes I and II loading-a statistical approach. *Theor. Appl. Fract. Mech.* **69**, 17–25 (2014).

[13] Khan, K. & Al-Shayea, N. A. Effect of specimen geometry and testing method on mixed mode I-II fracture toughness of a limestone rock from Saudi Arabia. *Rock Mech. Rock Eng.* **33**(3), 179–206 (2000).

[14] Pietras, D., Aliha, M. R. M., Kucheki, H. G. & Sadowski, T. Tensile and tear-type fracture toughness of gypsum material: Direct and indirect testing methods. *J. Rock Mech. Geotech. Eng.* **15**(7), 1777–1796 (2023).

[15] Paraskevopoulou, C. *et al.* The three stages of stress relaxation-observations for the time-dependent behaviour of brittle rocks based on laboratory testing. *Eng. Geol.* **216**, 56–75 (2017).

[16] Guessous, Z., Gill, D. E. & Ladanyi, B. Effect of simulated sampling disturbance on creep behaviour of rock salt. *Rock Mech. Rock Eng.* **20**, 261–275 (1987).

[17] Schapery, R. A. Nonlinear viscoelastic and viscoplastic constitutive equations with growing damage. *Int. J. Fract.* **97**, 33–66 (1999).

[18] Bérest, P., Blum, P. A., Charpentier, J. P., Gharbi, H. & Valès, F. Very slow creep tests on rock samples. *Int. J. Rock Mech. Min. Sci.* **42**(4), 569–576 (2005).

[19] Hamza, O. & Stace, R. Creep properties of intact and fractured muddy siltstone. *Int. J. Rock Mech. Min. Sci.* **106**, 109–116 (2018).

[20] Roberts, L. A., Buchholz, S. A., Mellegard, K. D. & Düsterloh, U. Cyclic loading effects on the creep and dilation of salt rock. *Rock Mech. Rock Eng.* **48**, 2581–2590 (2015).

[21] Sinkala, P. *et al.* Creep damage model for rockburst at Mufulira mine in Zambia. *Min. Metall. Explor.* **39**, 1983–2000 (2022).

[22] Heap, M. J., Baud, P. & Meredith, P. G. Influence of temperature on brittle creep in sandstones. *Geophys. Res. Lett.* **36**(19), 563 (2009).

[23] Bandeira, M. V. V., La Torre, K. R., Kosteski, L. E., Marangon, E. & Riera, J. D. Influence of contact friction in compression tests of concrete samples. *Constr. Build. Mater.* **317**, 125811 (2022).

[24] Shah, K. R. & Labuz, J. F. Damage mechanisms in stressed rock from acoustic emission. *J. Geophys. Res. Solid Earth* **100**(B8), 15527–15539 (1995).

[25] Přikryl, R., Lokajíček, T., Li, C. & Rudajev, V. Acoustic emission characteristics and failure of uniaxially stressed granitic rocks: The effect of rock fabric. *Rock Mech. Rock Eng.* **36**, 255–270 (2003).

[26] Maranini, E. & Brignoli, M. Creep behaviour of a weak rock: Experimental characterization. *Int. J. Rock Mech. Min. Sci.* **36**(1), 127–138 (1999).

[27] Günther, R. M., Salzer, K., Popp, T. & Lüdeling, C. Steady-state creep of rock salt: Improved approaches for lab determination and modelling. *Rock Mech. Rock Eng.* **48**, 2603–2613 (2015).

[28] Brace, W. F., Paulding, B. W. & Scholz, C. Dilatancy in the fracture of crystalline rocks. *J. Geophys. Res.* **71**, 3939–3953 (1966).

[29] Heap, M. J., Baud, P., Meredith, P. G., Bell, A. F. & Main, I. G. Time-dependent brittle creep in Darley Dale sandstone. *J. Geophys. Res.-Solid Earth.* **114**, B07203 (2009).

[30] Zhu, W. C., Li, S. H., Li, S. & Niu, L. L. Influence of dynamic disturbance on the creep of sandstone: An experimental study. *Rock Mech. Rock Eng.* **52**, 1023–1039 (2019).

[31] Kim, J. S., Lee, K. S., Cho, W. J., Choi, H. J. & Cho, G. C. A comparative evaluation of stress-strain and acoustic emission methods for quantitative damage assessments of brittle rock. *Rock Mech. Rock Eng.* **48**, 495–508 (2015).

[32] Sivakumar, G. & Maji, V. B. Crack growth in rocks with preexisting narrow flaws under uniaxial compression. *Int. J. Geomech.* **21**(4), 04021032 (2021).

[33] Xue, Y. C. *et al.* Time-dependent cracking and brittle creep in macrofractured sandstone. *Int. J. Rock. Mech. Min. Sci.* **162**, 105305 (2023).

[34] Taheri, A., Yfantidis, N., Olivares, C. L., Connelly, B. J. & Bastian, T. J. Experimental study on degradation of mechanical properties of sandstone under different cyclic loadings. *Geotech. Test. J.* **39**(4), 673–687 (2016).

[35] Tra, V., Kim, J. Y., Jeong, I. & Kim, J. M. An acoustic emission technique for crack modes classification in concrete structures. *Sustainability* **12**(17), 6724 (2020).

[36] Polyzos, D., Papacharalampopoulos, A., Shiotani, T. & Aggelis, D. G. Dependence of AE parameters on the propagation distance. *J. Acoust. Emiss.* **29**, 57–67 (2011).

[37] Aggelis, D. G., Mpalaskas, A. C. & Matikas, T. E. Investigation of different fracture modes in cement-based materials by acoustic emission. *Cem. Concr. Res.* **48**, 1–8 (2013).

[38] Farhidzadeh, A., Mpalaskas, A. C., Matikas, T. E., Farhidzadeh, H. & Aggelis, D. G. Fracture mode identification in cementitious materials using supervised pattern recognition of acoustic emission features. *Constr. Build. Mater.* **67**, 129–138 (2014).

[39] Zhu, W. C., Li, S., Niu, L. L., Liu, K. & Xu, T. Experimental and numerical study on stress relaxation of sandstones disturbed by dynamic loading. *Rock Mech. Rock Eng.* **49**, 3963–3982 (2016).

[40] Yang, S. Q. *et al.* Deformation and damage failure behavior of mudstone specimens under single-stage and multi-stage triaxial compression. *Rock Mech. Rock Eng.* **52**, 673–689 (2019).

[41] Scavia, C. A method for the study of crack propagation in rock structures. *Géotechnique.* **45**(3), 447–463 (1995).

[42] Wang, C. L., Zuo, C. & Zhao, Z. Evolution model of coal failure using energy dissipation under cyclic loading/unloading. *Appl. Sci.* **13**, 5797 (2023).

[43] Chen, L., Jia, B. X. & Zhang, S. G. Study on mechanical behavior and energy mechanism of sandstone under chemical corrosion. *Materials.* **15**, 1613 (2022). 35208152 10.3390/ma15041613 PMC8879642

[44] Ganne, P., Vervoort, A. & Wevers, M. Quantification of pre-peak brittle damage: Correlation between acoustic emission and observed micro-fracturing. *Int. J. Rock Mech. Min. Sci.* **44**(5), 720–729 (2007).

[45] Zhao, J. J. *et al.* A study of acoustic emission and damage evolution of limestone under different stress paths and confining pressures. *Geofluids.* **2021**, 6165366 (2021).

[46] Zafar, S., Hedayat, A. & Moradian, O. Micromechanics of fracture propagation during multistage stress relaxation and creep in brittle rocks. *Rock Mech. Rock Eng.* **55**, 7611–7627 (2022).

